# Biosensor for ATP detection via aptamer-modified PDA@POSS nanoparticles synthesized in a microfluidic reactor

**DOI:** 10.1007/s00604-024-06186-7

**Published:** 2024-02-23

**Authors:** Güneş Kibar, O. Berkay Şahinoğlu, Betül Kılınçlı, E. Yegan Erdem, Barbaros Çetin, V. Cengiz Özalp

**Affiliations:** 1 Dept. Materials Sci. & Eng., A.T. Adana Sci. & Tech. Uni., Adana, 01250 Turkey; 2 Microfluidics & Lab-on-a-chip Research Group, İ.D. Bilkent Uni., Ankara, 06800 Turkey; 3 UNAM-National Nanotech. Research Center and Inst. Materials Sci. & Nanotech., İ.D. Bilkent Uni., Ankara, 06800 Turkey; 4 Dept. Mech. Eng., İ.D. Bilkent Uni., Ankara, 06800 Turkey; 5 Dept. Food Eng., A.T. Adana Sci. & Tech. Uni., Adana, 01250 Turkey; 6 Dept. Medical Biology, School of Medicine, Atılım Uni., Ankara, 06836 Turkey

**Keywords:** Biosensor, Polyhedral oligomeric silsesquioxane (POSS), Aptasensor, Microfluidics, ATP detection

## Abstract

This study introduces aptamer-functionalized polyhedral oligomeric silsesquioxane (POSS) nanoparticles for adenosine triphosphate (ATP) detection where the POSS nanoparticles were synthesized in a one-step, continuous flow microfluidic reactor utilizing thermal polymerization. A microemulsion containing POSS monomers was generated in the microfluidic reactor which was designed to prevent clogging by using a continuous oil flow around the emulsion during thermal polymerization. Surfaces of POSS nanoparticles were biomimetically modified by polydopamine. The aptamer sequence for ATP was successfully attached to POSS nanoparticles. The aptamer-modified POSS nanoparticles were tested for affinity-based biosensor applications using ATP as a model molecule. The nanoparticles were able to capture ATP molecules successfully with an affinity constant of 46.5 $\upmu$M. Based on this result, it was shown, for the first time, that microfluidic synthesis of POSS nanoparticles can be utilized in designing aptamer-functionalized nanosystems for biosensor applications. The integration of POSS in biosensing technologies not only exemplifies the versatility and efficacy of these nanoparticles but also marks a significant contribution to the field of biorecognition and sample preparation.

## Introduction

Nucleic acid aptamers, including DNA, RNA, or modified nucleic acids, are artificially designed molecules that are capable of adopting three-dimensional structures which allow them to selectively bind to targets with high affinity and sensitivity at comparable levels to antibodies [1, 2]. The selection of aptamers is conducted in vitro using a sophisticated combinatorial methodology known as Systematic Evolution of Ligands by Exponential Enrichment (SELEX). This process involves the iterative screening and amplification of a vast library of nucleic acid sequences to identify those with the highest affinity for the target molecule [1, 3]. The SELEX method stands out for its ability to rapidly evolve highly selective aptamers, making it a powerful tool in both research and therapeutic applications. Through multiple rounds of binding, partitioning, and amplification; SELEX efficiently identifies aptamers with optimal binding characteristics, resulting in molecules that are finely tuned to their targets with high affinity and selectivity that are often comparable to, or even surpass, those of traditional antibodies. This advanced technique has significantly broadened the scope of aptamer applications, establishing them as a formidable class of biomolecules in the fields of molecular biology, diagnostics, and therapeutic development, as well as adenosine triphosphate (ATP) detection.

ATP is the primary molecule responsible for carrying energy in cellular metabolism and its depletion in the myocardial cells is related to the diseased state in cardiovascular pathologies. The monitoring of ATP in cardiac metabolism of heart failure conditions has substantial therapeutic importance [4] and in general, the necessity for ATP detection in cell biology research is increasing [5]. Following the pioneering work of Huizenga et al. [6] on identification of aptamers for the recognition of ATP, several studies utilized aptamers for ATP detection [6–11] as well as discrimination between ATP, adenosine diphosphate (ADP) and adenosine monophosphate (AMP) [12, 13] utilizing different adsorbent types such as columns [6, 7, 11], magnetic beads [10] and nanoparticles [8, 9, 12].

Polyhedral oligomeric silsesquioxane (POSS)-based materials have emerged as innovative hybrid monolithic columns, gaining importance in sample preparation and high-performance liquid chromatography (HPLC) separations. This advancement is attributed to organic-inorganic hybrid nature with an affinity to easily accept different radical groups, versatile material properties such as heat resistance, biocompatibility and easy modification of the carbon content [14–18]. A notable development in this field is the enhancement of specific recognition capabilities of monolithic columns. This enhancement has been achieved through the integration of molecular imprinting strategies and aptamer recognition techniques with POSS, a concept extensively explored in [19, 20]. In several studies, aptamer-POSS hybrid monoliths were utilized in affinity chromatography techniques to detect food toxins [21–23]. In addition, Hong et al. [24] synthesized aptamer-modified POSS-perovskite quantum dots (POSS-PQDs-Apt) as signal probe and titanium carbide (Ti3C2) MXenes as a quencher to detect *Vibrio parahaemolyticus*. Moreover, the versatility of POSS extends to the development of drug delivery systems [25]. In addition to Fluorescence Resonance Energy Transfer (FRET) biosensors, there have been significant advancements in the development of colorimetric, fluorescent, and printed electronic-based aptasensors incorporating POSS [26, 27]. These developments show the potential of POSS in biosensing and sample preparation applications; especially POSS in the nanoparticle form emerges as an important advancement in biodetection.

Nanoparticles have frequently been used with aptamers for improved biosensor development due to extremely high surface-area-to-volume [28]. Among them, silica nanoparticles have several advantages that make them a suitable platform for aptamer-based assays such as convenient surface modification and favorable biocompatibility, and have been implemented for ATP detection [8, 9]. Alternative to silica-based materials, POSS-based materials present a feasible alternative due to their hybrid structure. Although there has been some effort on aptamer-modified POSS-based biosensors, there is still a need for an aptamer-based ATP detection using POSS nanoparticles, offering the advantage of straightforward handling with conventional laboratory equipment, eliminating the need for sophisticated tools like HPLC or a fluorescence spectrometer. In this study, we employ aptamers for the detection of ATP molecules by utilizing POSS nanoparticles. Synthesis within a controlled environment to obtain desired shape and size distribution is a key ingredient to achieve a sensitive detection with POSS nanoparticles. Therefore, we have utilized a microfluidic reactor in the synthesis process.

Some batch methods were developed to synthesize POSS nanoparticles [29–31]. For example, in a recent study [30], POSS micro/nanoparticles were synthesized in one-step emulsion polymerization using different ultrasound sources (ultrasonic bath and prob-sonication) and surfactants in batch. Even though the one-step polymerization worked properly to obtain spherical nanoparticles, a significant effect of utilizing ultrasound sources was not observed on the synthesis results. While batch methods are widely accepted and understood for micro/nanoparticle synthesis, microfluidic approaches or microreactors present numerous benefits, including, but not limited to, high-throughput analysis, the utilization of minimal reactant volumes, and precise regulation of reaction parameters such as temperature and concentration, thereby yielding monodisperse particle shape and size [32–36]. In a microfluidic system, the reaction itself is constrained to a specific volume, consequently restricting the size of the end product [37, 38] and leads to a decrease in the reaction’s time constant, attributed to faster sample heating and cooling [35]. These distinctive properties of microfluidic reactors make them an attractive method for conducting controlled chemical reactions. Previously, the production of hybrid micro/nano particles within microreactors were reported in literature [21, 33, 36, 39–42]. On the other hand, the synthesis of POSS particles is a relatively recent aspect in this field, and nearly all reported research on their synthesis has employed batch techniques.

**Fig. 1:**
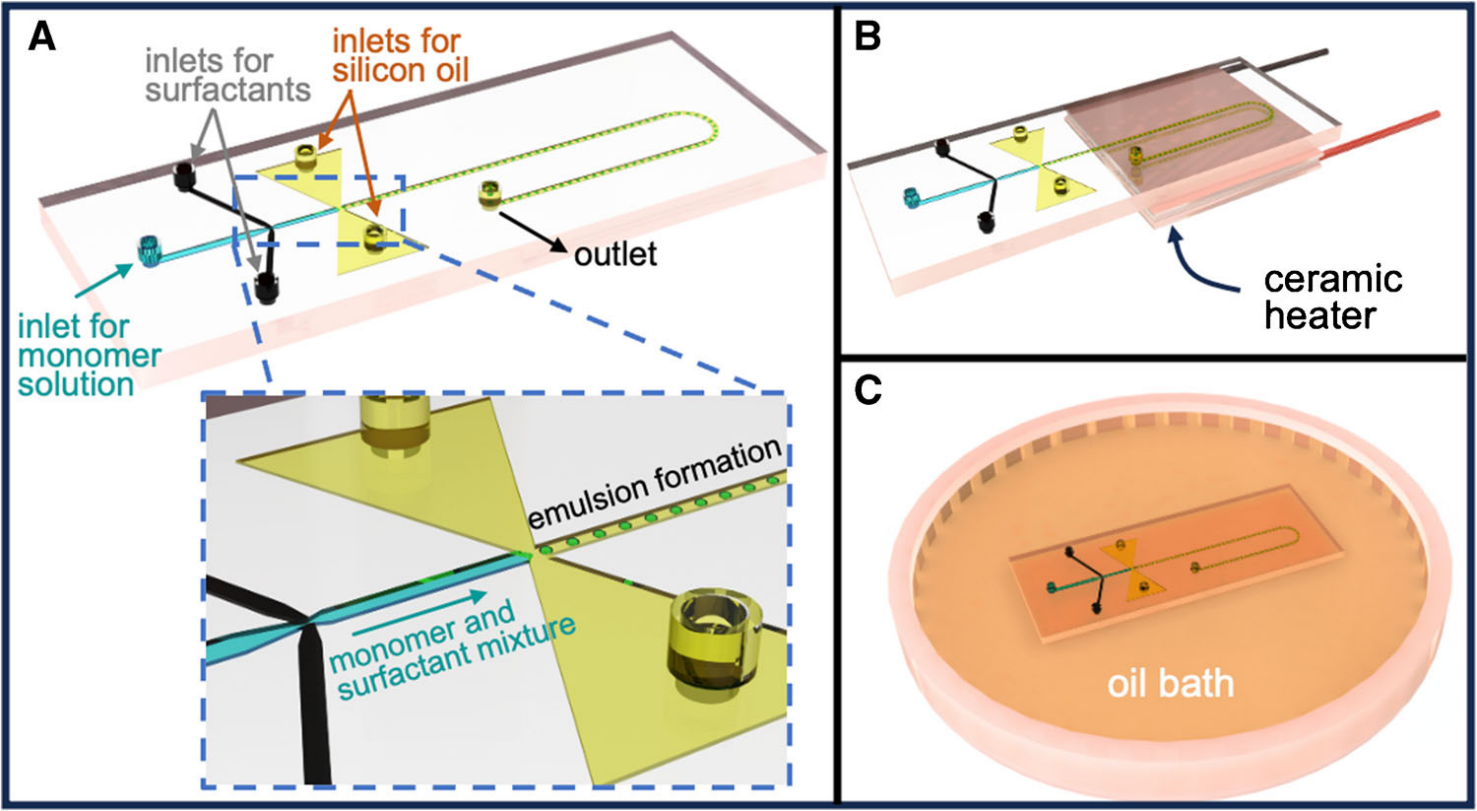
Schematics of the microfluidic device for the synthesis of POSS nanoparticles: **(A)** Monomer and surfactant phases create an emulsion at the first mixing junction. Experimental schematics of the microfluidic reactor with **(B)** a Peltier heater and **(C)** an oil bath

Our group has recently presented the one-pot synthesis of spherical, monodisperse POSS microparticles within a continuous flow microfluidic reactor featuring two distinct temperature zones [43]. This method relied on dispersion polymerization and produced uniform-sized microparticles, while the same composition of dispersion medium resulted in POSS nanoclusters in batch-wise production. However, after thermal polymerization, the microfluidic reactor had a clogging problem due to the accumulation of POSS microparticles onto microchannel surfaces, which leads to a reduction in product yield [43]. To overcome this issue, a microfluidic reactor which enables one-step synthesis of POSS nanoparticles by creating emulsion through continuous flow and utilizing thermal polymerization is developed. To the best of the authors’ knowledge, this is the first study in the literature that presents POSS nanoparticle synthesis via one-step emulsion polymerization without any ultrasound sources in a microfluidic reactor. A microemulsion (50–300 nm droplets) containing POSS monomers was generated by self-assembly in the continuous flow microfluidic reactor. The reactor was specially designed with the double mixing unit to solve the clogging problems in the microchannel during thermal polymerization. Surfaces of POSS nanoparticles were biomimetically modified by polydopamine. The covalent attachment of ATP aptamer sequences onto the surface of POSS nanoparticles was performed, followed by the characterization of their capture efficiency towards ATP molecules. Cytidine triphosphate (CTP) was utilized to demonstrate the preferential binding of the aptamer-modified POSS nanoparticles to ATP over other non-target nucleotides. In conclusion, this is a pioneering work that reports the use of aptamer-functionalized, microfluidically synthesized POSS nanoparticles for ATP detection.

## Materials and method

### Fabrication of the microfluidic reactor

The microfluidic channel was designed to allow active mixing of polymer medium within double emulsion segments in a continuous flow as shown in Fig. 1A. The channel dimensions were 100 $\upmu$m$\times$400 $\upmu$m (height and width). The microfluidic reactor was fabricated by using the standard soft lithography technique. First, a 100 $\upmu$m thick SU-8 mold (SU8-2050, Microresist Technology, Germany) was fabricated on a silicon wafer (prime (100)-N type wafer, Nanografi) using photolithography. Then, PDMS (Sylgard 184 Polydimethylsiloxane, Dow Corning, USA) oligomer was mixed with the crosslinking agent in weight ratio of 10:1. This mixture was poured onto the SU-8 mold and cured at 80^∘^C for 90 min. Once the PDMS mold was cured, it was peeled from the master mold and the channel inlets and outlets were punched out. As a final step, a glass slide was treated in a tabletop atmospheric plasma cleaner (Harrick Plasma Cleaner, Ithaca, NY, USA) and bonded on the PDMS mold to seal the microchannels.

**Fig. 2:**
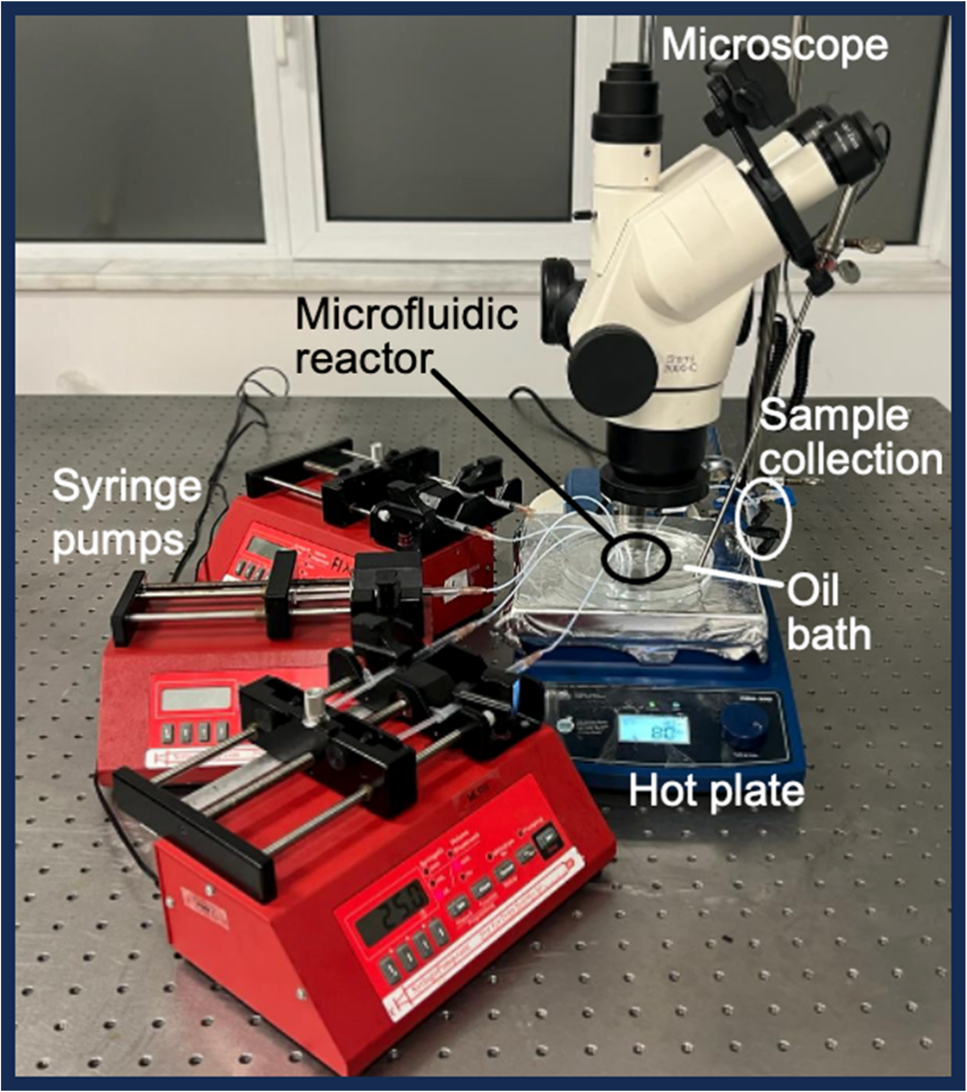
Experimental setup of the microfluidic synthesis of POSS nanoparticles

**Table 1:** Summary of the experimental conditions for the synthesis of POSS nanoparticles

Monomer phase	
M-POSS (monomer)	0.20 [g]
AIBN (thermal initiator)	0.02 [g]
EtOH (solvent)	2.0 [mL]
Flow rate	4.0 [$\upmu$L/min]
Surfactant phase	
SDS (Surfactant)	0.30 [g]
Water (Solvent)	5.0 [mL]
Flow rate	2.0 [$\upmu$L/min]
Oil phase	
Silicon oil	2.0 [mL]
Flow rate	2.0 [$\upmu$L/min]

### Synthesis of POSS nanoparticles

The POSS nanoparticles were synthesized using emulsion polymerization with thermally initiated free radical mechanisms in the microfluidic reactor. The pre-emulsion solutions, containing monomer, surfactant, and the oil phase, were prepared in advance before being introduced into the reactor. The monomer 0.2 g of Methacryl-POSS (Hybrid Plastics Inc. MA-0735), thermal initiator 0.02 g of 2,2^′^-Azobisizobutyronitrile (AIBN, Sigma-Aldrich), and the monomer solvent 2 mL of absolute ethanol (ETOH, Sigma-Aldrich) were mixed as the monomer phase. The surfactant 0.3 g of sodium dodecyl sulfate (SDS) was dissolved in 5 mL of deionized (DI) water (H2O, Millipore/Direct Q-3UV) to obtain the surfactant phase. A silicon oil (1000 cSt Sigma-Aldrich) was utilized as the oil phase. As seen in Fig. 2, the experimental configuration included syringe pumps responsible for dispensing the oil phase, monomers, and surfactants. The monomer phase was loaded into a 2 mL syringe (Beybi, TR) and pumped into the reactor from the main inlet with a flow rate of 4 $\upmu$L/min. The surfactant solution was split into two portions, placed into 2 mL syringes and introduced from the side inlets at the first mixing junction with a flow rate of 2 $\upmu$L/min each. The oil phase was loaded into a 2 mL syringe and introduced from the side inlets at the second mixing junction with a flow rate of 2 $\upmu$L/min. The experimental conditions are summarized in Table 1. After the second mixing junction, the emulsion was heated to 70^∘^C to polymerize the POSS nanoparticles. The entire procedure was under constant observation through a camera. For the heating step, two approaches were assessed for the control of heating. A Peltier unit (TEC1-4905) with 17 W heating capacity was positioned directly beneath the microfluidic channel to maintain the reaction temperature (as illustrated in Fig. 1B). Alternatively, the microfluidic reactor was submerged in a hot oil bath (a conventional tool in every research lab) placed on a hot plate (as illustrated in Fig. 1C). Following the polymerization, the resulting POSS nanoparticles were collected at the outlet of the reactor. The collected sample was centrifuged at 15,000 rpm for 2 min and was subsequently washed twice with ethanol to eliminate any residual oil and reaction medium. The collected particles were dried at 50^∘^C in a vacuum oven overnight. For both heating approaches, it was observed that the reproducibility of the emulsion and the characteristics of POSS nanoparticles were quite similar; however, it was also observed that the yield was increased by approximately 30% in the case of heating in an oil bath. Therefore, submerging in a hot oil bath was implemented for the rest of the study.

### Polydopamine coating of POSS nanoparticles

The POSS nanoparticles were coated with polydopamine (PDA) as described by Kibar et al. [31]. 0.25 mg of synthesized POSS nanoparticles were dispersed in 2 mg/mL dopamine hydrochloric (DOPA-HCl, Sigma-Aldrich) containing 10 mL of Tris-Buffer (pH 8.5). The mixture was magnetically stirred for 6 h in a dark hood. The reaction solution changed the color from white to dark. After PDA coating, POSS nanoparticles were collected via centrifugation at 15,000 rpm for 2 min and washed with DI water several times. The collected particles were dried at 50^∘^C in a vacuum oven overnight.

### Aptamer decoration of POSS nanoparticles

5^′^ amino labelled ATP aptamer (5^′^-CCACCACGGTGGTG GTGGTTGTGGTGC-3^′^) were obtained from Oligomer Ltd. (Ankara, Türkiye) [12]. The PDA@POSS nanoparticles were modified to carboxyl functionalized via a EDC linker (1-ethyl-3-(3-dimethylaminopropyl carbodiimide hydrochloride, Sigma-Aldrich). Basically, PDA@POSS nanoparticles were prepared to a concentration of 0.1 $\upmu$g/$\upmu$L by adding 50 mM EDC and 20 mM NHS (N-hydroxy succinimide, Sigma-Aldrich), and mixing at room temperature for 1 h. The particles were collected via centrifugation at 15,000 rpm for 2 min. 300 $\upmu$L of aptamer (1 $\upmu$M) was added to particles and incubated overnight for the binding. Then, the Aptamer@PDA@POSS (Apt-POSS) nanoparticles were collected via centrifugation at 15,000 rpm for 2 min and washed by phosphate-buffered saline (PBS, pH 7.4) to remove unbind-aptamer content.

### Characterization of POSS nanoparticles

The morphology and size of the particles were analyzed by scanning electron microscopy (SEM, Quanta 450 Akishima, Tokyo, Japan). The SEM images were evaluated for the size distribution of the POSS nanoparticles using a Java-based image processing program, ImageJ (National Institutes of Health, USA). The size distribution histogram was obtained using ORIGIN (2021), a computer program for interactive scientific graphing and data analysis. The chemical structure of the particles was determined by Fourier transform infrared spectroscopy FTIR (Thermo Scientific Nicolet^TM^, USA).

**Fig. 3:**
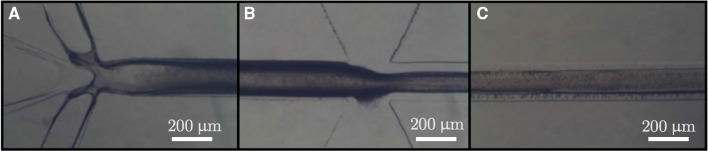
Microfluidic synthesis of POSS nanoparticles: **(A)** Monomer and surfactant phase creates an emulsion at the first mixing junction, **(B)** oil phase focuses the stream at the second mixing junction, and **(C)** polymerization occurs during the flow through the microfluidic channel. The microscope images at 10$\times$ magnification using inverted microscope (PSARON-FluidoScope)

### ATP detection

An ATP binding assay was based on the determination of ATP molecules by measuring absorption at 260 nm in a UV-VIS Spectrophotometer (Omega, BMG Labtech). A series of ATP samples of 1 $\upmu$M to 2 mM were prepared and mixed with 0.01 mg of Apt-POSS nanoparticles, incubated for 30 min, and then centrifuged at 15,000 rpm for 2 min to separate bound and unbound ATP. Parallel experiments were also performed for CTP under the same experimental conditions, and all measurements were repeated three times.

The equilibrium binding of a single ligand species, which is ATP in our case, can be described by a form of Langmuir isotherm as [44]:

1 $$\begin{aligned} {q} = \frac{q_mC}{K+C} \end{aligned}$$

where *C* [$\upmu$M] is the equilibrium concentration of ATP, *q* is the amount of adsorbed ATP, $q_m$ is the maximum value of adsorbed ATP, and *K* [$\upmu$M] is the affinity constant. The adsorbed amounts are quantified by absorption at 260 nm. Therefore, the adsorption model can be written as:

2 $$\begin{aligned} \left( \frac{A}{A_{m}}\right) = \left( \frac{q}{q_{m}}\right) = \frac{C}{K+C} \end{aligned}$$

where *A* [a.u.] is the absorption at specific concentration and $A_m$ [a.u.] is the absorption which corresponds to maximum adsorption. Nonlinear curve fitting is performed by the built-in function lsqcurvefit, which utilizes Levenberg-Marquardt algorithm, available in MATLAB®. The regression coefficient is defined as:

3 $$\begin{aligned} R^2 = 1-\frac{\sum \limits _{j} (A_{j}-{A}_{\text {predicted}})^2}{\sum \limits _{j}(A_{j}-\bar{A})^2} \end{aligned}$$

where $\bar{A}$ is the mean of the $A_{j}$ data.

## Results and discussion

The POSS nanoparticles were synthesized in a continuous flow microfluidic reactor. The emulsion was generated with monomer and surfactant phase at the first mixing junction shown in Fig. 3A. The micromixing effect within the microchannel led to the spontaneous self-assembly of sub-micron-sized micelles. The emulsion consisting of 50–300 nm droplets, continued to flow through the microchannel until it reached the second junction, where the intention was to adjust the flow and focus the emulsion within the microchannel (Fig. 3B). This focusing ensured the prevention of channel clogging during the polymerization process and lead to an uninterrupted flow for the rest of the microchannel (Fig. 3C). In previous studies, POSS nanoparticles were typically produced through emulsion polymerization in a batch reactor, where an ultrasonic source was used to generate an emulsion [30]. The microfluidic reactor used in this work introduced the benefit of generating self-assembled emulsions without an external source and allowing for a continuous and well controlled synthesis.

**Fig. 4:**
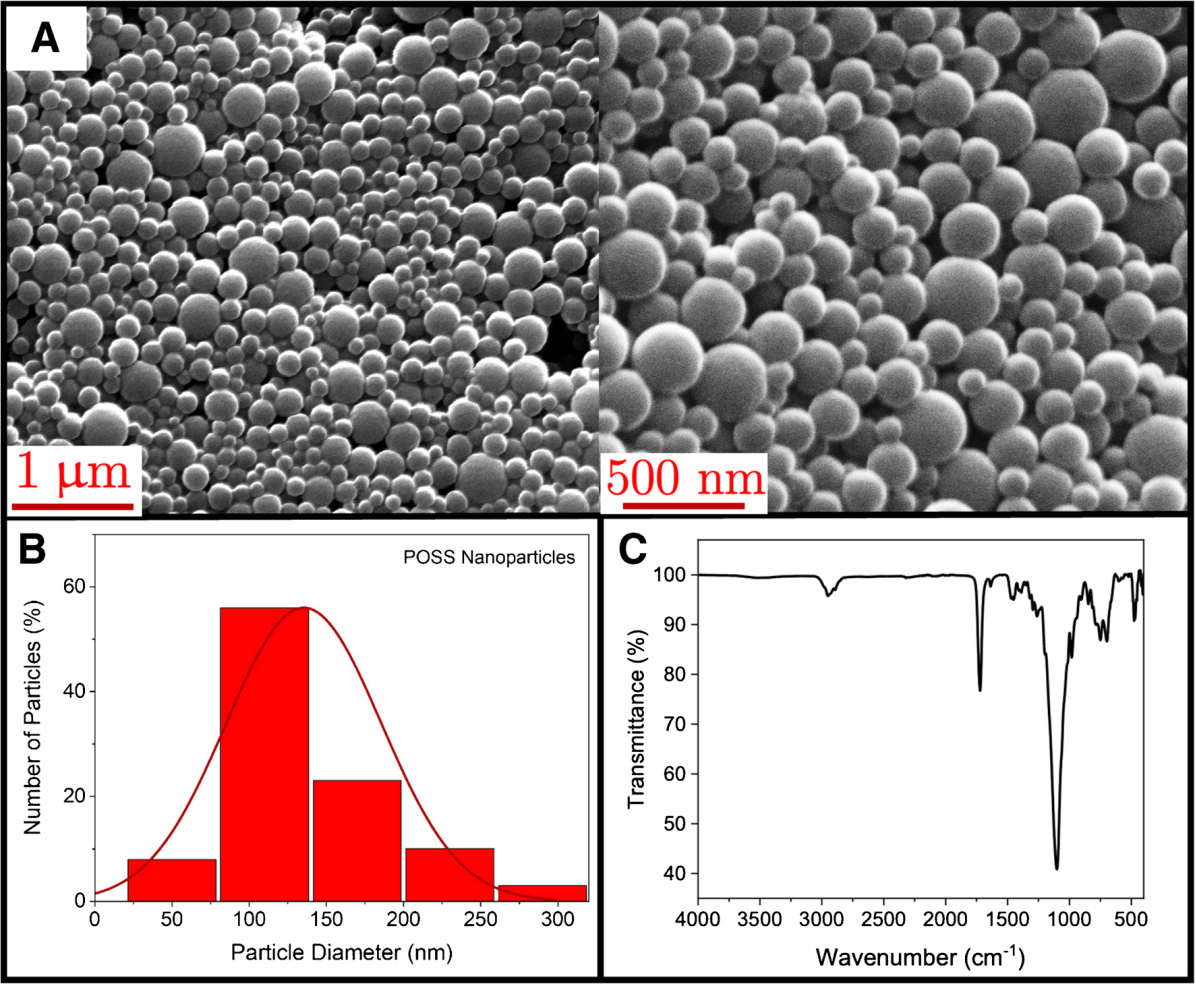
Characterization of POSS nanoparticles: **(A)** Morphological structure of POSS nanoparticles (SEM images at 50,000$\times$ and 100,000$\times$ magnification with 1 $\upmu$m and 500 nm scale bars, respectively), **(B)** size distribution histogram and **(C)** chemical structure (FTIR spectrum) of POSS nanoparticles

The particles were morphologically and chemically characterized. The SEM images show that the POSS nanoparticles were obtained in uniform spherical shape in an average sub-micron size of 140 nm (Fig. 4A and B). The small size enables rapid interactions with ATP molecules due to the high surface-to-volume ratio of POSS nanoparticles. FTIR spectrum reveals hybrid chemical structure with organic and inorganic content (Fig. 4C). The peak at 1100 cm^-1^ represents $\text {Si}-\text {O}-\text {Si}$ inorganic cage structure [45, 46]. The peaks of the organic part were for the C=O stretching at 1730 cm^-1^, $\text {CH}_{3}$ $-\text {CH}_{2}$ stretching at 2950 cm^-1^ and 2890 cm^-1^ [45, 46]. The hybrid organic-inorganic structure of POSS nanoparticles can be easily functionalized with various molecules such as antibodies, aptamers, or peptides to bind ATP selectively. The flexibility in functionalization allows for the customization of POSS nanoparticles for specific detection needs.

Green synthesis methods were employed for the surface modification of the POSS nanoparticles. A thin PDA layer was applied to coat the POSS nanoparticles. This biomimetic surface modification introduced catecholamine functional groups, which were subsequently utilized for EDC-NHS coupling to bind the aptamers.

**Fig. 5:**
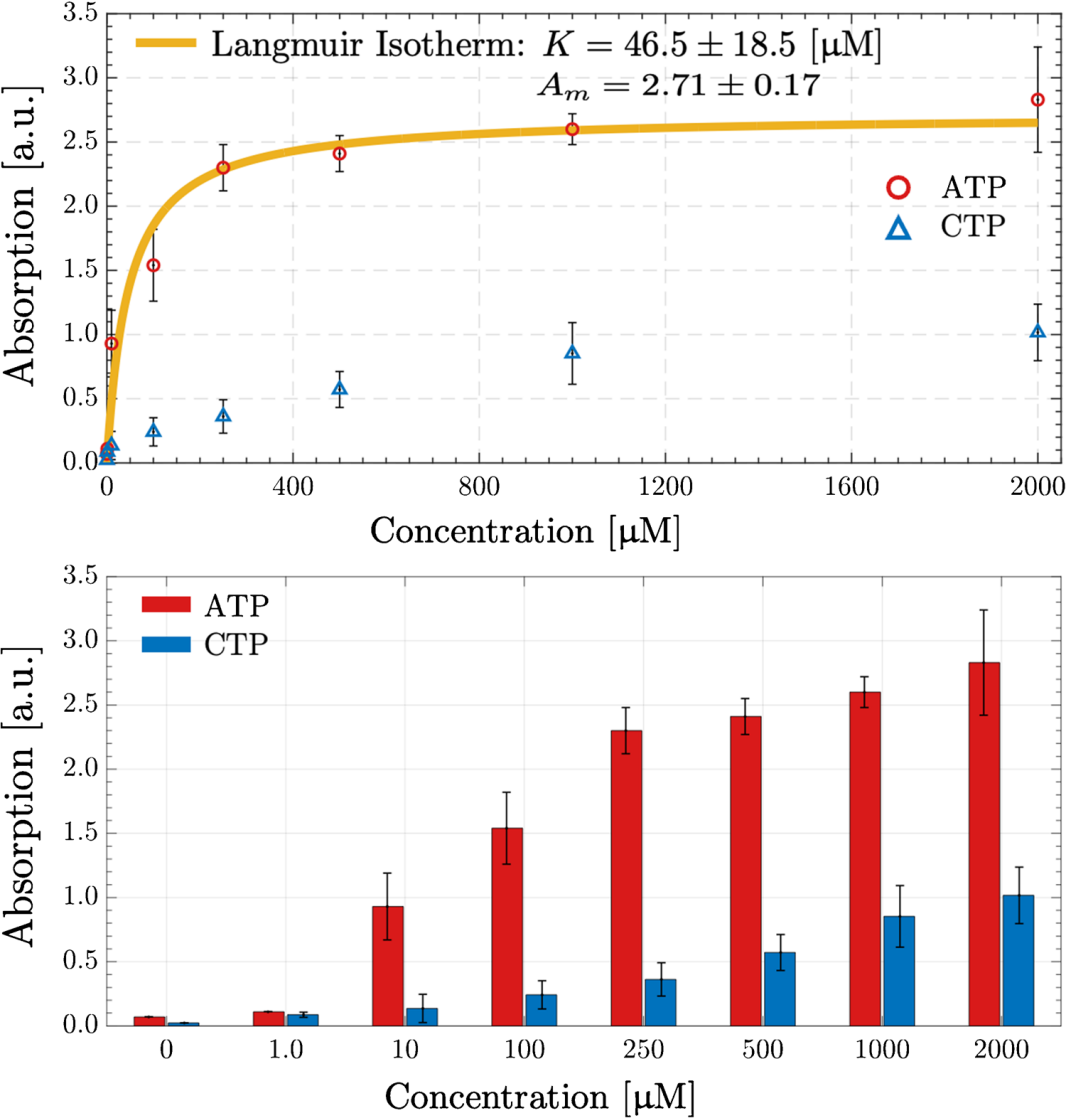
Absorption results for ATP and CTP. The values are mean with ± standard error from three independent repeated experiments at each concentration

**Table 2:** Summary of different studies regarding ATP detection available in the literature

Aptamer type	Adsorbent	Method	Affinity constant (K)
DNA aptamer [6]	Agarose column	Analytical	6 $\upmu$M
		ultrafiltration	
RNA aptamer [7]	RNA-bonded	Column	5 $\upmu$M
	column	elution assay	
DNA aptamer [8]	Silica	Fluorescent	34 $\upmu$M
	nanoparticles	(Cy5) assay	
DNA aptamer [9]	Silica	Fluorescent	1.0 mM
	nanoparticle	(hoechst33258)	
DNA aptamer [10]	Magnetic microbeads	Cytometric assay	$\sim$5 mM
DNA aptamer [11]	Streptavidin	Column	230 nM
	agarose resin		
DNA aptamer [12]	Polyacrylamide	Fluorescent	700 $\upmu$M
	nanoparticle	assay	
DNA aptamer	PDA@POSS	Spectrometric	46.5 $\upmu$M
(this study)	nanoparticles	assay	

Aptamers have a remarkable capability of binding to their target molecules. This specificity in binding is attributed to their three-dimensional structure, which is intricately influenced by the nucleotide composition and spatial positioning of the oligonucleotide molecule [47]. A distinctive feature of these aptamers is their ability to be identified and selected through an in vitro process, a method that allows for the identification of specific binding sequences from a combinatorial library. Notably, their affinity constants are often found to be comparable to those of antibodies, indicating the high specificity and efficiency of aptamers in target molecule recognition. For ATP binding, several aptamer sequences have been successfully selected, with their affinity constants predominantly situated within the low micromolar range [48], proving the precision and effectiveness of aptamers in binding to ATP. Furthermore, these aptamers have been meticulously selected for their preferential binding to ATP over other non-target nucleotides such as guanosine triphosphate (GTP), CTP, thymidine triphosphate (TTP), and uridine triphosphate (UTP). The efficacy of the Apt-POSS particles in capturing the target molecule ATP was evaluated by assays using spiked samples in phosphate-buffered saline (PBS). Absorption data at 260 nm were used to monitor the ATP molecules in experiments containing different concentrations of ATP. CTP was utilized to demonstrate the exceptional selectivity of the Apt-POSS particles. This use of CTP as a non-target molecule in the study serves to highlight the specificity of the Apt-POSS particles in distinguishing and binding to their intended target, ATP, thereby emphasizing the potential applications of aptamer-based technologies in selective molecular recognition and binding. Figure 5 illustrates the relationship between the absorbance of ATP captured by Apt-POSS particles and the ATP concentration in the buffer. The results for CTP are also included in the figure. CTP molecules are not the target of aptamers, and are used as control experiments for specificity. The CTP molecules were also captured by the Apt-POSS particles but at considerably lower amounts. The results indicate that Apt-POSS particles exhibit a high efficacy in capturing ATP molecules from the solution. It can be clearly seen that the amount of ATP and aptamer binding is dependent upon the concentration of ATP and conforms to a binding curve that exhibits saturation. The adsorption model and binding curve for ATP and Apt-POSS followed a one-site saturation ligand binding with an affinity constant of 46.5±18.5 $\upmu$M for 0.1 mg particle/mL concentration. This finding is notable when compared to a range of reported ATP binding aptamer sequences, which exhibit affinity constants ranging from millimolar to sub-micromolar levels, as summarized in Table 2. Although different studies used different aptamer sequences, the affinity constant (*K*) may be employed for the performance comparison among different assays. The lower the affinity constant, the higher the specific binding between aptamer and ATP molecules. In this sense, the affinity constant of column-based assays has a higher performance than that of ours, which comes with a price of integration of sophisticated tools like HPLC. Compared to the studies with micro/nanoparticles, our affinity constant has a comparable performance with that of Wang et al. [8], and is superior to others [9, 10, 12]. The performance improvement over Qu et al. [10] can be attributed to the micron scale of the magnetic particles. Our study can be directly compared to that of [12] since both studies implemented the same aptamer sequence. In the study of Özalp et al. [12], the affinity constant was determined to be 700 $\upmu$M via a measurement through surface plasmon resonance analysis following the immobilization of the aptamer sequence on a gold surface. Notably, the immobilization of the aptamer sequence on POSS nanoparticles resulted in a significant enhancement of its affinity constant, leading to an improvement of approximately 15-fold.

## Concluding remarks

This study introduces a novel aptamer-based ATP detection method that utilizes POSS nanoparticles synthesized in a microfluidic reactor that facilitates the one-step synthesis through the creation of an emulsion followed by thermal polymerization. Surfaces of POSS nanoparticles were biomimetically modified by PDA. The aptamer series for ATP was successfully attached to POSS nanoparticles. The Apt-POSS nanoparticles were tested for affinity-based biosensor applications using ATP as a model molecule. Thus, the Apt-POSS nanoparticles were used to capture ATP molecules specifically with 46.5 $\upmu$M affinity constant in the use of 0.1 mg particle/mL concentration. The result of this study reveals that POSS nanoparticles synthesized in a microfluidic reactor can be employed to design aptamer-functionalized nanosystems for biosensor applications.

The organic and inorganic nature of POSS nanoparticles brings new opportunities for different advanced material applications due to their hybrid nature. In addition, the organic groups may further allow surface modifications of the POSS nanoparticles with some functional groups such as antibodies, fluorescent molecules, silver nanoparticles, gold nanoparticles, or magnetic nanoparticles, which may lead to new generation detection (chromatography, SERS, SPR, etc.) or catalysis systems. Moreover, the inorganic silica cage structure of POSS nanoparticles exhibits biocompatibility, thermal degradation at high temperatures, as well as enhanced mechanical properties, which are suitable for applications such as dental materials, nanofillers, composite additives, or thermal insulation. Even though the current microfluidic synthesis of POSS nanoparticles has a limited yield, it can be improved by the implementation of parallel microfluidic reactors which will be one of our future research directions

## Data Availability

The data set used and analyzed during the current study are available from the corresponding author on reasonable request.
